# Accelerated Development of Novel Biomass-Based Polyurethane Adhesives via Machine Learning

**DOI:** 10.1021/acsami.4c20371

**Published:** 2025-02-28

**Authors:** Ye Cheng, Takuma Araki, Naofumi Kamimura, Eiji Masai, Masaya Nakamura, Sergei Manzhos, Tsuyoshi Michinobu

**Affiliations:** a Department of Materials Science and Engineering, Institute of Science Tokyo, 2-12-1 Ookayama, Meguro-ku, Tokyo 152-8552, Japan; b Department of Forest Resource Chemistry, Forestry and Forest Products Research Institute, Tsukuba, Ibaraki 305-8687, Japan; c Department of Materials Science and Bioengineering, Nagaoka University of Technology, Nagaoka, Niigata 940-2188, Japan; d Department of Chemical Science and Engineering, Institute of Science Tokyo, 2-12-1 Ookayama, Meguro-ku, Tokyo 152-8550, Japan

**Keywords:** adhesive strength, Bayesian optimization, biomass, machine learning, polyurethane

## Abstract

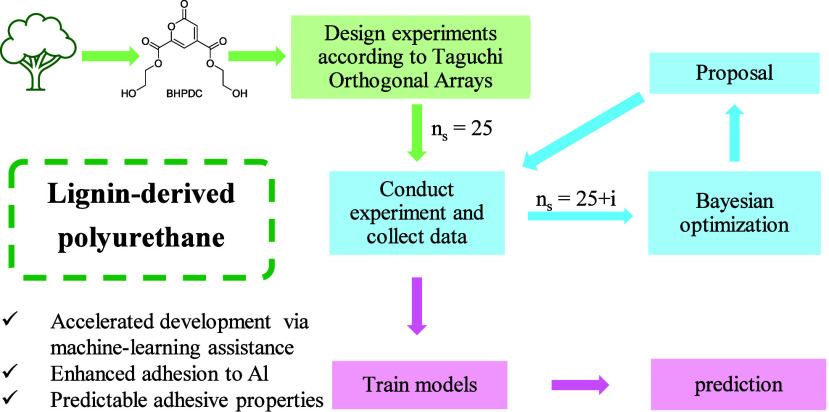

2-Pyrone-4,6-dicarboxylic acid (PDC) can be produced on a large scale from lignin by transformed bacteria, and its use as a bifunctional monomer to synthesize biomass-based polymers has been reported. Recently, excellent adhesive properties of the resulting biomass-based polymers were also reported, but their performance has not yet been optimized. In this study, we focus on improving the adhesive properties of PDC-based polyurethanes (PUs) by combining experiments and machine learning (ML). We synthesized an initial data set of 25 adhesive samples from different polyols and isocyanates with different isocyanate-to-hydroxyl ratios (*r*). Adhesive strengths were measured after hot-pressing at varying temperatures (*T*_heat_, °C) and durations (*t*_heat_, h), following a Taguchi L25 orthogonal design. Gaussian process-based Bayesian optimization (BO) was employed to identify an optimal PDC-based PU adhesive as a function of polyol type, isocyanate type, *r* ratio, heating temperature, and time with an improved adhesive strength of 10.04 ± 1.26 MPa after only five iterations. This approach highlights the effectiveness of BO in guiding experimental conditions for an enhanced performance. Random Forest regression was also used as an alternative ML approach and supported the conclusions. Overall, this study demonstrates the potential of the BO in accelerating the development and optimization of novel adhesive materials.

## 1 Introduction

In recent decades, increasing attention has been focused on biomass-based polymers rather than those derived from petroleum because of their potential to be produced in a sustainable and environment-friendly way.^1−3^ Lignin is one of the most abundant natural carbon resources, which is metabolized from plants and bacteria.^4−6^ During microbial degradation of lignin, a stable intermediate, 2-pyrone-4,6-dicarboxylic acid (PDC), is naturally produced by *Sphingobium* sp. SYK-6 before being incorporated into the tricarboxylic acid cycle.^7^ Otsuka et al. first successfully produced PDC on a large scale,^8^ and later they managed to isolate pure PDC by fractional precipitation with Na^+^ ions,^9^ strengthening the case for subsequent studies on its practical application.^10−18^ In previous reports, PDC-based polymers were found to show attractive adhesive properties to different substrates such as Al, brass, Cu, Fe, SUS304, glasses, and wood.^19−25^ Given the trend toward replacing petroleum-derived products with biomass-derived products, further development of the application of PDC-based polymers as strong adhesives is of great significance.

Conventional materials development usually requires considerable time and effort not only because it requires prior experience and accumulated expertise in chemistry, physics, and materials but also because it incurs substantial equipment, manpower, and consumable costs.^26−28^ In recent decades, the superior performance of machine learning (ML) in deriving structure/conditions–property relations from large and complex data sets have led to an increasing interest in its application in a variety of fields.^29−31^ Applying ML to the field of materials, and specifically to assist in the design of materials, which is also called materials informatics, has attracted more and more attention in recent years.^32−42^ One of the main goals of ML in materials development is to find specific materials with outstanding performance in a shorter time and at a lower cost. For computation-guided materials design, Gaussian process regression (GPR)-based Bayesian optimization (BO) has recommended itself as particularly promising, including for the development of epoxy resins and adhesives.^43−47^

One major obstacle in the field of materials informatics in general and in the present work focusing on the design of PDC-based polymers is limited data availability, as there are still relatively few examples of studies on PDC-based adhesives. Data-based methods work best with large amounts of data which sometimes can be sourced from literature or generated within a project.^48^ One often has to work with relatively few data, which is a challenge for ML for which various workarounds are being researched.^49,50^ Furthermore, there are many factors other than the structure of the polymer itself that affect adhesive strengths, such as the type of substrate, surface treatment of the substrate, adhesion measurement conditions, and experiment environment. Consequently, data on PDC-based adhesives reported by different studies may be noisy and inconsistent under different experimental conditions, making it difficult to obtain accurate data sets. Hence, we decided to conduct experiments by ourselves in this study to obtain an initial data set under the same experimental environment.

Recent developments in ML have significantly minimized the challenge of “small data”, enabling models to perform well even when trained on limited data sets.^51−54^ For instance, Bone et al. effectively used hierarchical ML to predict high-precision bioprinting with alginate, using a data set of just 48 prints to optimize material selection, formulation, and process variables for specific constructs.^55^ Similarly, Ming et al. utilized a Random Forest model with only 40 data points to discover new Mn^4+^-doped fluoride red phosphors with desirable excited-state lifetimes through an iterative process.^56^ In this study, given the novelty of PDC-based polymers, we employed Gaussian process-based BO to iteratively expand our small initial data set in a way that is biased toward higher adhesive strength polymers.

Polyurethane (PU) is one of the commonly used adhesives, and its properties can be adjusted by polyols and isocyanates with different ratios of hard and soft segments.^57^ The hard segments in PU are formed from the reaction of isocyanates with chain extenders or low-molecular-weight diols, which provide rigidity and contribute to the mechanical strength and thermal stability of the adhesive. Conversely, the soft segments are usually long-chain polyols, imparting flexibility and elasticity to the material. By varying the ratio of hard to soft segments, the adhesive properties of PUs can be finely tuned to achieve the desired balance of strength and flexibility, making them versatile for various applications.^58−60^ PU-based adhesives have been extensively studied and applied in various fields due to their excellent mechanical properties, chemical resistance, and adaptability to different substrates. For example, Zhang et al. developed a solvent-free and highly cross-linked nonisocyanate PU adhesive with strong adhesion (20.67 MPa), substrate versatility, and environmental tolerance, including resistance to ultralow temperature (−196 °C) and organic solvents, which demonstrated potential for critical applications in aerospace, energy, and chemical industries.^61^ Similarly, Rusen et al. investigated the biopolyol products obtained from broadleaf sawdust being used in six different PU adhesive formulations for wood bonding, which increased the glass transition temperatures, enhanced the stiffness, and reduced the brittleness of PUs, valorizing biomass waste into products with added value for indoor applications.^62^ Furthermore, Wang et al. synthesized a series of biobased PUs with excellent adhesion properties from eco-friendly polyester diols with different lactic acid/ε-caprolactone feed ratios, demonstrating the potential applications in electronic appliances, the automotive industry, and construction materials.^63^

In a previous study, we demonstrated that PDC-based PUs showed excellent adhesion to various substrates (Al, Cu, and Fe).^23^ In this study, we focus on using ML to accelerate the development of PDC-based PUs with improved adhesive properties. In order to obtain the initial data set, we first prepared 25 samples of PDC-based PU adhesives from the combination of different polyols and isocyanates at the different isocyanate-to-hydroxyl ratio, *r*. Their adhesive strengths to metal substrates were measured after hot-pressing at different heating temperatures, *T*_heat_ (°C), and heating times, *t*_heat_ (h), according to the experimental conditions proposed by the Taguchi L25 orthogonal design.^64,65^ Starting from the very small initial data set, we then used BO to search for the optimized adhesive strength of PDC-based PUs prepared under different experimental conditions. Guided by the ML model, we conducted additional experiments on most promising predicted materials, iteratively expanding the data set size, with BO applied after each iteration. After five cycles, a PDC-based PU with an excellent adhesive property was obtained. Finally, we used several regression models to train the final data set obtained after five iterations of BO, producing an ML model that is able to provide a reliable estimate of adhesive strength that can be used to guide future experiments. We think this approach can accelerate the development of new adhesive materials, especially when initial data sets are very small, while simultaneously reducing experimental time and cost.

## 2 Materials and Methods

### 2.1 Materials

PDC was prepared from protocatechuate via the metabolic pathway of *Sphingobium* sp. SYK-6, as reported previously.^8^ Bis(2-hydroxyethyl)-2-oxo-2*H*-pyran-4,6-dicarboxylate (BHPDC) was synthesized according to a previous report.^66^ Diphenyl phosphate (DPP), methylenediphenyl 4,4′-diisocyanate (MDI), dicyclohexylmethane 4,4′-diisocyanate (HMDI), and isophorone diisocyanate (IPDI) were purchased from Tokyo Kasei Kogyo Co.; 1,5-pentamethylene diisocyanate (PDI) was supplied from Mitsui Chemicals, Inc.; and lysine diisocyanate (LDI) was supplied from Chuo Kaseihin Co. These chemicals were used as received without further purification. Poly(tetrahydrofuran) 2000 (PTHF2000) and polyethylene glycol 2000 (PEG2000) were purchased from Tokyo Kasei Kogyo Co., polypropylene glycol 2000 (PPG2000) was purchased from Sigma-Aldrich Japan Co., andpolylactic acid 2000 (PLA2000) was supplied from HighChem Co.; these chemicals were dried in a desiccator at 60 °C for over 24 h before use. ε-Caprolactone (ε-CL) was purchased from Tokyo Kasei Kogyo Co. and was distilled before use. Polycaprolactone 2000 (PCL2000) was synthesized according to a previous report.^67^ The chemical structures of polyols and diisocyanates are shown in Figure S1.

### 2.2 Synthesis of PDC-Based Polyurethanes

**Figure 1:**
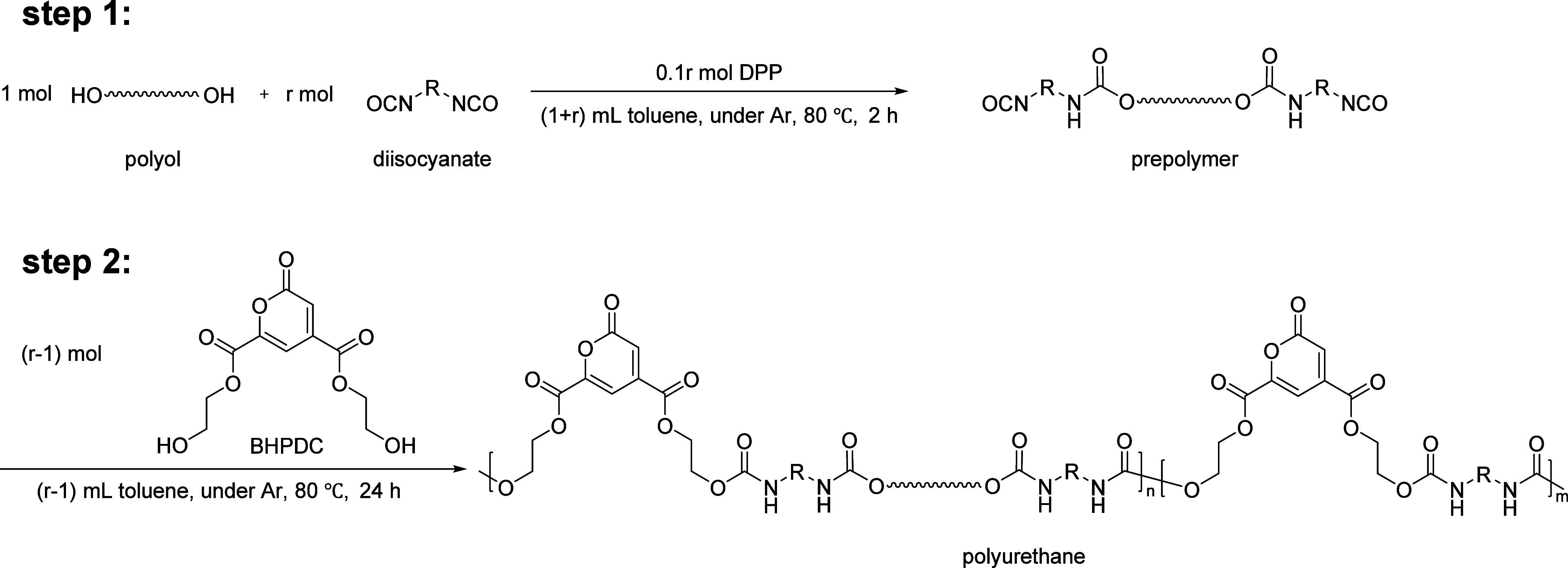
General Synthetic Scheme of PDC-Based Polyurethanes Using Bis(2-hydroxyethyl) 2-Oxo-2*H*-pyran-4,6-dicarboxylate (BHPDC).

The isocyanate-to-hydroxyl ratio *r* means that if 1 mol of polyol is used for synthesis, *r* mol of diisocyanate, 0.1 mol of DPP, and (1 + *r*) mL of toluene were used in the first step, and (*r* – 1) mol of BHPDC and (*r* – 1) mL of toluene were added in the second step (Figure 1). The five different polyols, isocyanates, and *r* used to synthesize PUs are summarized in Table 1 and were later used as independent input variables for the ML models. The five polyols were selected because of their potential biodegradability, and the five isocyanates were selected because they are widely used in PU synthesis and are commercially available. In particular, PDI and LDI are derived from biomass, which is more environmentally friendly with lower CO_2_ emissions than the other petroleum-based isocyanates. The values of *r* were chosen by considering the practicality of PU synthesis. A typical procedure for the synthesis of PU is as follows (taking No. 3 in Table S1 as an example): DPP (52.4 mg, 0.2094 mmol), PTHF2000 (1969.8 mg, 0.9849 mmol), LDI (424.8 mg, 2.0018 mmol), and 3 mL of toluene were added to a reaction vessel in an Ar-filled glovebox. After the reaction mixture was heated to 80 °C for 2 h, BHPDC (271.4 mg, 0.9970 mmol) and another 1 mL of toluene were added to the reaction mixture and stirred at 80 °C for another 24 h. After this, the reaction mixture was dissolved in CH_2_Cl_2_ and precipitated in cold methanol to give PU as a pale-yellow compound (2327.4 mg, 87.3%). δ_H_ (400 MHz, Chloroform-*d*): 7.53, 7.50, 7.16, 7.14, 7.10, 5.49, 5.27, 4.85, 4.50, 4.36, 4.29, 4.02, 3.71, 3.38, 3.12, 1.81, 1.76, 1.58, 1.48, 1.34, 1.22. IR(neat): ν = 3334, 2938, 2853, 2795, 1722, 1530, 1484, 1446, 1411, 1367, 1332, 1242, 1211, 1175, 1104, 1027, 984, 960, 893, 775, 763, 712, 690, 654, 613, 596, 580, 569, 553, 539, 522, 511 cm^–1^.

**Table 1:** Summary of Variable Parameters Used to Synthesize Polyurethane Adhesives for the BO

	variable parameters^a^				
no.	polyol	isocyanate	*r*	*T*_heat_ (°C)	*t*_heat_ (h)
1	PTHF	MDI	2	250	0.5
2	PEG	PDI	3	200	1
3	PPG	LDI	4	150	2
4	PCL	IPDI	5	100	3
5	PLA	HMDI	6	70	4

a Variable parameters include type of polyol, type of isocyanate, isocyanate-to-hydroxyl ratio *r*, heating temperature *T*_heat_ (°C), and heating time *t*_heat_ (h).

### 2.3 Characterization of the Adhesive Properties to Aluminum Substrates

For the single-lap shear test according to the JIS K 6850-1999 testing method, aluminum test pieces (A1050P-H24) with the size of 1.6 × 25 × 100 mm were used as substrates, and the measurements were done by an autograph AGS-10kNX (Shimadzu Co.). These substrates were successively washed with acetone and methanol for 10 min each, and the washed substrates were further treated with a UV-O_3_ cleaner (Filgen, Model UV253E) for 10 min before use. Approximately 30 mg of PU was then placed on one face of a pair of aluminum test piece surfaces (10 × 25 mm). A set of two plates was in contact with each other by hot-pressing at 10 MPa and cured at a specified temperature for a certain time under a vacuum. The values of heating temperature and heating time in Table 1 are initially adopted values for the preparation of adhesive samples and were later used as input data for the BO. To be specific, five heating temperatures and times were chosen according to our previous knowledge that high temperature is needed to open the 2-pyrone ring of the PDC unit to coordinate to hydroxides and metal oxides on the metal surface.^22^ However, there must be optimized conditions because a longer heating time and lower temperature sometimes resulted in stronger adhesion. Therefore, it is meaningful to pursue the best combination between the heating temperature and heating time. The failure force of the PUs against the aluminum test pieces was measured at room temperature at a rate of 1 mm/min. The adhesive strengths were calculated as the failure force divided by the bonding area (10 × 25 mm). Each measurement was repeated for at least twice, and the average value was reported with the standard deviation (Table S1).

### 2.4 Design of Experimental Conditions for the Initial Data Set

The initial data set of *n*_s_ = 25 samples was designed according to the Taguchi Orthogonal Arrays. The Taguchi Array can produce uniform scatter data points such that for each level of a particular parameter, all levels of each other parameter are tested at least once.^64,65^ There are 3125 (5^5^) possible experimental conditions in this study that were provided by a combination of five types of polyols, five types of isocyanates, five isocyanate-to-hydroxyl ratios, five heating temperature *T*_heat_ (°C) values, and five heating time *t*_heat_ (h) values (Table 1). By adopting a Taguchi L25 orthogonal design, 25 experimental conditions were obtained (Table S1).

### 2.5 Machine Learning Methods

All data processing and ML algorithms were performed using Python (version 3.8.10). The BO was conducted by using GPyOpt (version 1.2.6) and GPy (version 1.12.0). The categorical variables (type of polyols and isocyanates) were converted into numerical variables via one-hot encoding, while the real-valued variables that are *r*, heating temperature, and heating time (Table 2) were scaled within the range from 0 to 1. Thus, a total of five features were used.

**Table 2:** Experimental Results of Samples Prepared under Various Conditions (Initial Dataset, Dataset Size *n*_s_ = 25 Samples)^a^

	variable parameters						
no.	polyol	isocyanate	*r*	*T*_heat_ (°C)	*t*_heat_ (h)	adhesive strength (MPa)	*T*_d,5%_ (°C)
1	PTHF	MDI	5	250	1	1.15 ± 0.06	273
2	PTHF	PDI	6	200	2	2.76 ± 0.03	243
3	PTHF	LDI	2	70	4	0.09 ± 0	308
4	PTHF	IPDI	3	150	3	2.38 ± 0.27	274
5	PTHF	HMDI	4	100	0.5	0.74 ± 0.01	273
6	PEG	PDI	5	70	3	0.06 ± 0.03	254
7	PEG	LDI	6	150	0.5	0 ± 0	250
8	PEG	IPDI	2	100	1	0.18 ± 0.17	273
9	PEG	HMDI	3	250	2	1.82 ± 0.75	263
10	PEG	MDI	4	200	4	6.52 ± 0.98	266
11	PPG	LDI	5	100	2	0.25 ± 0.11	276
12	PPG	IPDI	6	250	4	0 ± 0	262
13	PPG	HMDI	2	200	3	0.76 ± 0.05	285
14	PPG	MDI	3	70	0.5	0.16 ± 0.01	274
15	PPG	PDI	4	150	1	0.11 ± 0.03	274
16	PCL	IPDI	5	200	0.5	1.37 ± 0.55	242
17	PCL	HMDI	6	70	1	0.14 ± 0	242
18	PCL	MDI	2	150	2	0 ± 0	232
19	PCL	PDI	3	100	4	0.43 ± 0.02	196
20	PCL	LDI	4	250	3	1.75 ± 0.37	269
21	PLA	HMDI	5	150	4	1.18 ± 0.31	112
22	PLA	MDI	6	100	3	0.89 ± 0.04	125
23	PLA	PDI	2	250	0.5	0 ± 0	120
24	PLA	LDI	3	200	1	0 ± 0	156
25	PLA	IPDI	4	70	2	0.54 ± 0.04	134

a Variable parameters include type of polyol, type of isocyanate, isocyanate-to-hydroxyl ratio *r*, heating temperature *T*_heat_ (°C), and heating time *t*_heat_ (h). Experiments under each set of conditions were repeated twice to calculate the deviation.

### 2.6 Bayesian Optimization

BO is widely used to seek the best combination of variables that yields the best results. In this step, the search range was set to the same variable parameters as in Table 1, with 3125 possible experimental conditions. The acquisition function was set to Expected Improvement (EI), and the other specific settings are listed in Table S1. EI selects points that have a high probability of improving the objective function, thus efficiently guiding the search toward the optimum. Given the small initial data set, EI helped make the most out of each experiment by focusing on points that were expected to improve the adhesive strength significantly. By prioritizing points with a high EI, we reduced the number of experimental trials needed to find the optimal adhesive formulation, saving both time and resources. EI has been successfully applied in various studies and domains for optimization problems similar to ours,^68,69^ which gives us confidence in its applicability and effectiveness for optimizing the adhesive properties of PDC-based PUs. The BO used GPR with the Matérn 5/2 kernel. Grid search was performed with leave-one-out cross-validation (LOOCV) on the training data to tune the hyperparameters of the models to prevent overfitting and improve model performance (Table S1).

Following the suggestion of BO, we carried out an experiment in our laboratory to obtain new data and added this new measured adhesive strength to the initial data set. BO is supposed to improve predictions as new data is added. Therefore, BO was conducted again with a new data set of 26 samples, and a new set of most promising experimental conditions was predicted. This step was repeated five times, resulting in a data set of 30 samples, of which 5 were around the expected best adhesive property. See the Supporting Information for UMAP (Uniform Manifold Approximation and Projection) analysis of clustering patterns in the parameter space along BO cycles and explanation of termination after five cycles.

### 2.7 Machine Learning Algorithms for Regression Predictions

The initial data set (*n*_s_ = 25) and five additional samples obtained with the help of BO were combined together for training the final ML model. As the BO was GPR-based, this resulted in a GPR model. In addition, we explored several other algorithms including linear regression, gradient boost regression, and Random Forest regression (RF) that were trained on the final data set. The best results were obtained with RF, which also allows evaluating the relative importance of features. In this study, the data set was divided into 80% of the original data set as training data to tune the hyperparameters and 20% as test data to test the model with a fixed random state of 0 to ensure consistency. To optimize the hyperparameters, LOOCV was used, whereby the model was trained on 23 samples and validated on the remaining single sample. This process was repeated 24 times, ensuring that each sample was used for validation once. The LOOCV was scored by “neg mean squared error” to optimize the hyperparameters. Then, the coefficient of determination (*R*^2^), the root-mean-square error (RMSE), and mean absolute error (MAE) of the predicted and the measured values on both training and test data were used to evaluate these ML models. The model with the lowest RMSE and MAE and the highest test set *R*^2^ value was considered the best and was used to further predict the adhesive strength for all 3125 possible experimental conditions (Table 1).

## 3 Results and Discussion

### 3.1 Experimental Results from the Initial Data Set

**Figure 2:**
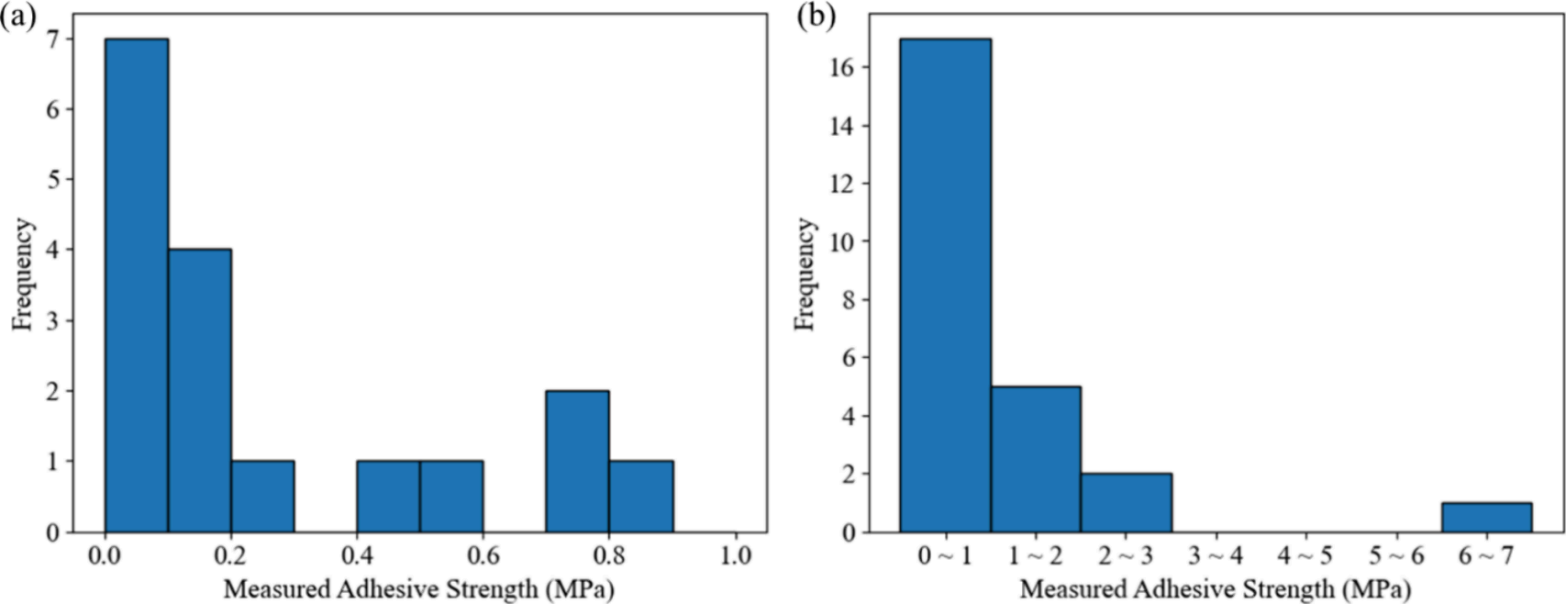
Distribution of adhesive strength (MPa) from the initial data set of size *n*_s_ = 25 samples (a) ranging from 0 to 1 MPa and (b) ranging from 0 to 7 MPa.

Experimental results of the adhesive strength (MPa) based on the Taguchi L25 orthogonal design are summarized in Table 2. These initial data sets were prepared under various conditions, including the type of polyol, the type of isocyanate, isocyanate-to-hydroxyl ratio *r*, heating temperature *T*_heat_ (°C), and heating time *t*_heat_ (h). Experiments under each set of conditions were repeated twice to calculate the deviations shown in the table. Figure 2 illustrates the distribution of adhesive strength (MPa) with an average of 0.93 and one-sigma deviation of 1.41 MPa. Approximately 68% of PUs exhibited a relatively weak adhesion strength of less than 1 MPa. However, a certain combination of parameters resulted in a comparatively strong adhesion of 6.52 MPa, which instilled confidence in our pursuit of an enhanced adhesion performance. Therefore, the BO was adopted to explore the possibility of improving adhesion.

### 3.2 Bayesian Optimization

**Figure 3:**
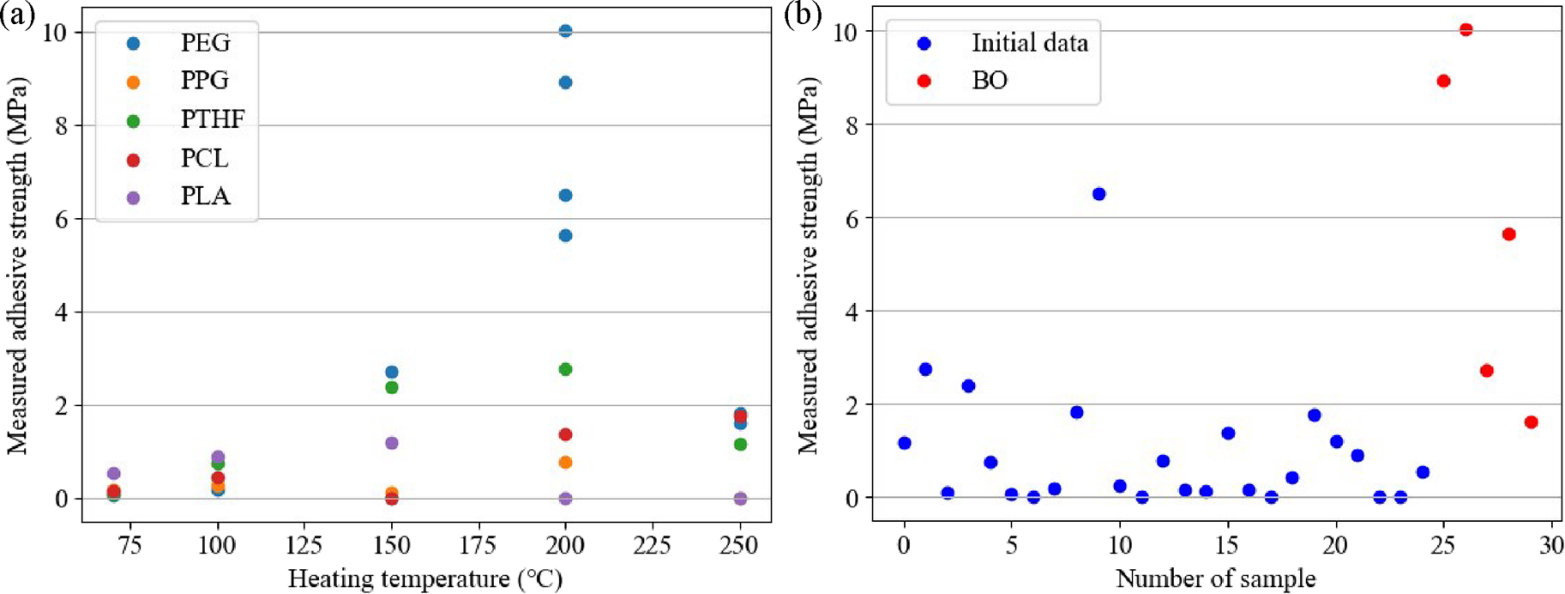
Distribution of adhesive strength (MPa) (a) grouped by different polyols at different temperatures (°C) and (b) from the initial data set alone (blue) and after BO (red).

After iteratively applying BO to the initial data set of 25 samples for five iterations as described above, the strongest adhesive strength was 10.04 MPa (No. 27 in Table 3). Compared with the previous study on PDC-based PU adhesives, whose maximum adhesive strength was 2.93 MPa,^23^ this adhesive strength can be considered as an extremely high value. From the suggestion for the next experimental conditions via BO, we noticed that the combination of PEG and MDI gave promising results in achieving a strong adhesion. The larger isocyanate-to-hydroxyl ratio *r*, which indicates more PDC units in the polymer chain, resulted in stronger adhesions. This is consistent with previous research reporting that the PDC unit exhibits strong adhesion to aluminum substrates.^19^ A possible mechanism for adhesion is the ring-opening reaction of the 2-pyrone ring at high temperatures and its coordination to hydroxides and metal oxides on the metal surface.^19^ Therefore, the longer the heating time, *t*_heat_ (h), the stronger adhesion can be achieved, which is consistent with BO suggestions. However, we also measured the decomposed temperatures of these PUs (Table 2), which are usually in the range from 230 to 270 °C except for PUs synthesized from PLA. The PLA-based PUs exhibited lower decomposition temperatures of 112 to 156 °C, causing no adhesion at 200 and 250 °C (Figure 3a). The strong adhesion requires a high curing temperature to open the 2-pyrone ring but should be below the decomposition temperature to prevent thermal decomposition of PUs. The heating temperature *T*_heat_ (°C) at 200 °C showed great improvement over a higher temperature of 250 °C or a lower temperature of 150 °C. This indicates that the right balance between the ring-opening temperature and PU decomposing temperature can be achieved by precisely adjusting the temperature with the help of BO.

**Table 3:** Proposed Preparation of Polyurethane Adhesives Predicted by BO and the Related Experimental Adhesive Strengths

	suggested experimental conditions						
no.	polyol	isocyanate	*r*	*T*_heat_ (°C)	*t*_heat_ (h)	predicted adhesive strength (MPa)	measured adhesive strength (MPa)
26	PEG	MDI	5	200	4	6.17 ± 0.25	8.94 ± 1.12
27	PEG	MDI	6	200	3	7.18 ± 1.68	10.04 ± 1.26
28	PEG	MDI	5	150	4	8.01 ± 0.83	2.71 ± 0.63
29	PEG	MDI	5	200	2	5.32 ± 4.44	5.64 ± 0.28
30	PEG	MDI	4	250	3	5.29 ± 3.83	1.6 ± 0.11

The distribution of adhesive strength measurements from the initial data set alone (blue) and after BO (red) is illustrated in Figure 3b. The adhesive strengths in the initial data set were concentrated in the range from 0 to 2.76 MPa except the No. 10 sample with the relatively high adhesive strength of 6.52 MPa. The spread of the data from the BO was wider than in the initial data set, and an outstandingly high adhesive strength of 10.04 MPa was obtained. These results confirm the potential of using ML for developing new adhesive materials when the number of data samples of relevant materials is limited, and a relatively high-performance result can be achieved in a few trials, considering the large number of combinations of all the free parameters involved. BO thus provides an efficient and intelligent method of assisting chemistry and materials researchers in achieving a better result in designing or optimizing structures and synthesis conditions.

### 3.3 Final ML Model

**Figure 4:**
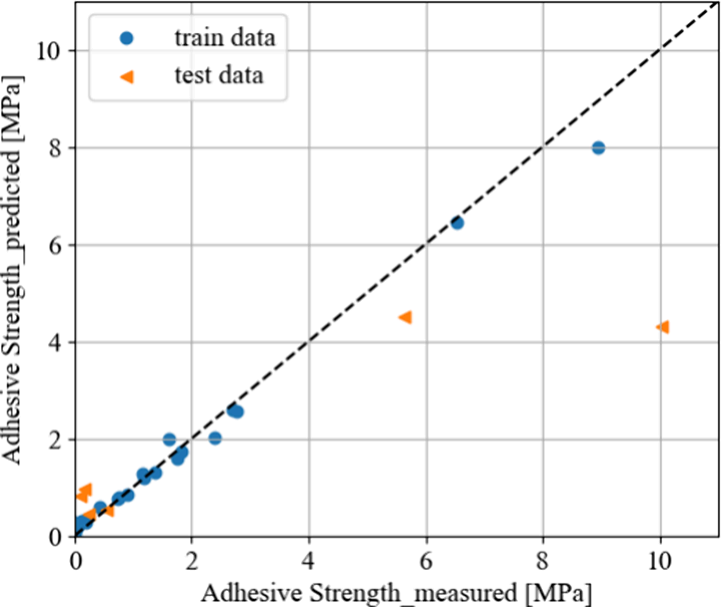
Predicted vs measured adhesive strengths (MPa) from single-lap shear tests via Random Forest. The dashed straight line indicates where the predicted and measured adhesive strengths are equal. Hyperparameters used are shown in Table S1.

Several ML algorithms were applied to the final BO-expanded data set to build the final ML model for adhesion strength prediction. Among the algorithms we tried, RF showed the best performance, with the results shown in Figure 4. The model resulted in train/test *R*^2^ values of 0.98/0.59, RMSE values of 0.26/2.43, and MAE values of 0.17/1.44. The results with other algorithms (including nonregularized and regularized linear regressions and gradient-boosted decision trees) are shown in the Supporting Information.

**Figure 5:**
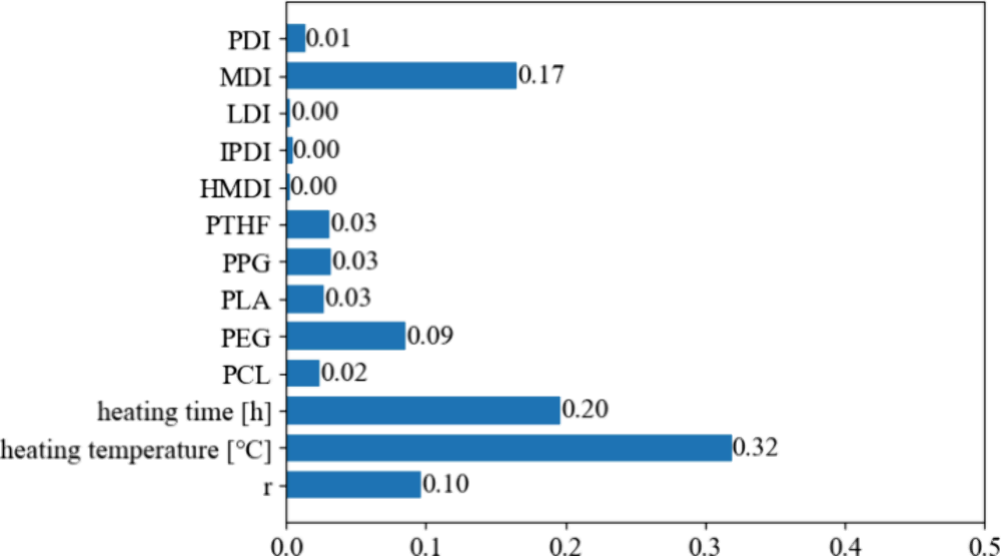
Feature importance in the Random Forest model.

The RF model also allows us to assess the relative importance of features, which is shown in Figure 5. The Random Forest model indicated that heating temperature and heating time were among the most important features, with importance scores of 0.32 and 0.20, respectively. The importance of heating temperature highlights the sensitivity of adhesive strength to thermal conditions, which is consistent with the conclusion from Section 3.2 that the right temperature is important to open the 2-pyrone ring of the PDC unit and coordinate to hydroxides and metal oxides on the aluminum surface, while at the same time the thermal decomposition of PUs must be considered because when the temperature was set at the highest value of 250 °C, the adhesive strength on the contrary decreased. Figure 5 also shows the importance of different polyols and isocyanates; while the ML features are the type of polyol and isocyanate, Figure 5 shows an effective explanatory power when a particular type is present. In the gradient boosted decision tree model (Figure S8d), features such as MDI (0.35) had the highest importance (0.36), followed by PEG (0.21) and heating time (0.21), suggesting that these components significantly enhance the adhesive properties. MDI is the only diisocyanate that has two aromatic rings in the molecule among the five candidate diisocyanates. Therefore, the PUs synthesized from MDI are expected to have increased stiffness and improved adhesive strength. PEG is formed by repeated linking units of ethylene glycol molecules, and in this case, PEG has more oxygen in the main chain than the other four polymers when the molecular weight is controlled at 2000. As a result, metal oxides are more likely to form on the aluminum surface, in turn increasing the adhesive strength. In addition, only PEG2000 and PLA2000 exhibit a solid state at room temperature, while the others are viscous liquids. As mentioned above, PLA-based PUs decomposed at relatively low temperatures, and accordingly, no strong adhesion was observed. Considering PEG2000 has a higher melting point than the other three liquid polymers, PEG-based PUs may crystallize at higher temperatures, which strengthens the mechanical and adhesive properties. Overall, it is crucial to choose a model that can strike a balance between the accuracy and interpretability of the ML models.

### 3.4 Random Forest Model for Predictions

We used the final ML model, RF, to predict the adhesive strength of all 3125 possible combinations of five experimental conditions. Table 4lists 10 conditions with the highest predicted adhesive strength. As shown in Table 4, PUs synthesized from PEG and MDI consistently yielded the highest adhesive strengths, indicating a favorable chemical environment for strong adhesive bonds. The highest adhesive strength of 8.38 MPa was achieved with an isocyanate-to-hydroxyl ratio (*r*) of 6, indicating that a higher ratio favors stronger adhesive bonds under the specified conditions. Ratios of 5 and 4 also resulted in significant adhesive strengths but to a lesser extent. This suggests that while high ratios are beneficial, there is flexibility in achieving good performance with lower ratios depending on other conditions. A critical hot-press temperature of 200 °C was identified, maximizing adhesion, while higher temperatures (e.g., 250 °C) led to lower strengths due to potential thermal degradation. The hot-pressing time showed a marginal but noticeable impact, with longer durations generally providing better results due to more complete adhesive curing. Our analysis also revealed that some features had minimal impact on the model’s predictions. This was evident from the feature importance scores, which indicated that variations in these features did not significantly affect the predicted adhesive strength values.

**Table 4:** Top 10 Adhesive Strengths Predicted by Random Forest According to 3125 Possible Combinations of Experimental Conditions

	variable parameters					
rank	polyol	isocyanate	*r*	*T*_heat_ (°C)	*t*_heat_ (h)	predicted adhesive strength (MPa)
1	PEG	MDI	6	200	4	8.38
2	PEG	MDI	6	200	3	8.22
3	PEG	MDI	5	200	4	7.55
4	PPG	MDI	5	200	3	7.39
5	PEG	MDI	6	200	2	6.81
6	PEG	MDI	4	200	4	6.69
7	PEG	MDI	4	200	3	6.60
8	PEG	MDI	6	250	4	6.23
9	PEG	MDI	5	200	2	6.04
10	PEG	MDI	4	200	2	5.70

**Figure 6:**
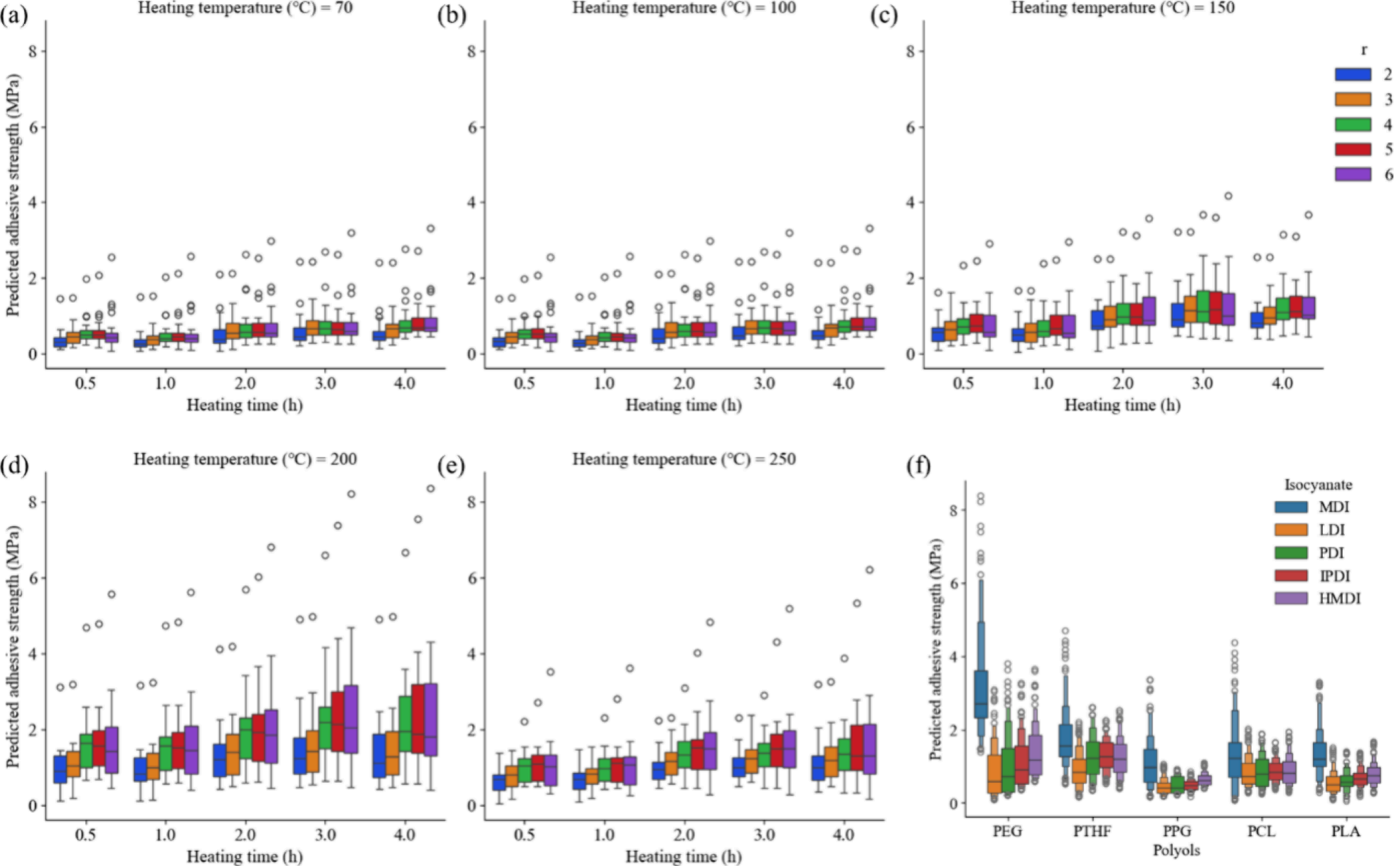
Predicted adhesive strengths (a–e) against heating time *t*_heat_ by isocyanate-to-hydroxyl ratio *r* at different heating temperatures *T*_heat_ and (f) against types of polyols by types of isocyanate.

All 3125 predicted adhesive strengths are illustrated in Figure 6. When the samples are prepared at temperatures lower than 200 °C, the resulting adhesive strengths remain in the low range of almost 2 MPa or less (Figure 6a–c). The ring opening of the 2-pyrone ring in the PDC unit occurs at temperatures higher than 200 °C, and coordination to hydroxides and metal oxides on the metal surface results in high adhesive strengths. However, the adhesive strength slightly decreases when hot pressing is performed at higher than 250 °C (Figure 6e). This slight decrease in the adhesive strength may originate from the decomposition of PUs at high temperatures, which is consistent with the experimental observations. As for the isocyanate-to-hydroxyl ratio *r*, it is clear that the adhesive strength significantly increases when *r* increases to 5 and 6 at 200 °C (Figure 6d) and 250 °C (Figure 6e), while at lower temperatures, the *r* value has no significant effect on the adhesive strength (Figure 6a–c). This may be evidence that the PDC unit is essential for improving the adhesive strength since higher *r* means a higher PDC content in the PU polymers. In the case of heating time, a slight increase in the adhesive strength is observed when hot press is conducted for a longer time. This increase can be attributed to the increased occurrence of the 2-pyrone ring in the PDC ring opening with longer heating times.

We also explored the influence of different polyols and isocyanates (Figure 6f). The adhesive strengths of PEG-based or MDI-based PUs spread in a wider range than the other PUs. This result suggests an opportunity to develop the PDC-based PUs with strong adhesion using PEG and MDI by tuning other experimental conditions.

Table 5 shows the predicted and measured adhesive strengths under various conditions, highlighting the model’s strengths and limitations in predicting adhesive strength under varying experimental conditions. While the model performs well for certain parameter combinations (Nos. 31, 34, and 35), deviation in No. 32 indicates the model’s limitations in accurately predicting adhesive strength at lower *r* and difference in No. 33 suggests that higher temperatures and extended heating times introduce complexities not fully captured in the model. Notably, when the heating temperature is 250 °C, a longer heating time inversely decreases the adhesive strength (Nos. 33 and 34), which is the opposite result of the model predictions. This may result from thermal degradation overwhelming the adhesion bonding at 250 °C over an extended heating period. Future work should focus on refining the model to account for these variables more accurately and consider additional data points to enhance the predictive accuracy. Overall, this analysis shows the importance of validating model predictions with an independent data set, and its reliability across diverse conditions can be improved via iteratively adding new data points to the model.

**Table 5:** Adhesive Strengths Predicted by Random Forest and the Experimental Adhesive Strengths

	variable parameters						
no.	polyol	isocyanate	*r*	*T*_heat_ (°C)	*t*_heat_ (h)	predicted adhesive strength (MPa)	measured adhesive strength (MPa)
31	PEG	MDI	6	200	1	5.62	7.11
32	PEG	MDI	5	200	1	4.85	6.56
33	PEG	MDI	6	250	3	5.20	3.03
34	PEG	MDI	6	250	2	4.84	5.33
35	PEG	MDI	6	250	1	3.62	4.90

## 4 Conclusions

The BO algorithm was employed to optimize the experimental conditions, including the type of polyol, type of isocyanate, isocyanate-to-hydroxyl ratio (*r*), heating temperature (*T*_heat_, °C), and heating time (*t*_heat_, h), to achieve the maximum adhesive strength of PDC-based PUs. This approach led to a significantly high adhesive strength of 10.04 ± 1.26 MPa, achieved from just 25 initial measurements through refining experimental conditions in five cycles of BO. This study highlights the efficiency of BO in accelerating the development of novel materials while reducing experimental time and costs, particularly when data collection is challenging due to limited literature.

Moreover, our ML models provided insights into optimizing the adhesive properties of PDC-based PUs. Specifically, PDC-based PUs synthesized from PEG and MDI with a high isocyanate-to-hydroxyl ratio (*r*) showed strong adhesion when hot pressed at 200 °C. Although the hot-pressing time had a marginal effect, longer durations still contributed to higher adhesive strength. Importantly, achieving strong adhesion requires balancing the ring-opening temperature of the 2-pyrone ring in PDC with the decomposition temperature of PDC-based PUs.

The insights gained from this study provide a clear pathway for synthesizing PDC-based PUs with a high adhesive strength. The identified optimal conditions can be used as starting points for further exploration and refinement. To enhance scalability and applicability, one can gather and analyze data across a broader spectrum of experimental conditions and material compositions, improving our methodology’s robustness. Future research can extend these findings by exploring other polyols with different molecular weights and various types of isocyanates and applying the optimized adhesives to different substrates. Moreover, beyond adhesion, other material properties, such as mechanical strength, heat resistance, and biodegradability, can also be targeted for optimization using ML approaches. In addition, further characterization of the high-adhesive-strength PDC-based PUs obtained in this study is essential to understanding the origins of their excellent properties and to guide researchers in developing and designing new high-performance PDC-based polymers. By addressing these avenues, it must be possible to establish a versatile framework for applying ML in the discovery and optimization of next-generation materials, thereby advancing industrial applications and understanding structure–property relationships.

## Supporting Information Available

Chemical structures of polyols and diisocyanates, NMR and IR spectra, the hyperparameters used in ML, and ML results (PDF)
Adhesive strengths predicted by Random Forest and the experimental adhesive strengths (ZIP)
