# Supplementary material for: Accelerated Development of Novel Biomass-Based Polyurethane Adhesives via Machine Learning
Source: ACS Appl Mater Interfaces. 2025 Feb 28;17(10):15959–68. doi: 10.1021/acsami.4c20371 (PMC11912195; doi:10.1021/acsami.4c20371)
Supplement: Supplementary file 1 — am4c20371_si_001.pdf [file am4c20371_si_001.pdf]

Supporting Information

Accelerated Development of Novel Biomass-based Polyurethane Adhesives via Machine Learning

Ye Cheng[a], Takuma Araki[b], Naofumi Kamimura[c], Eiji Masai[c], Masaya Nakamura[b], Sergei Manzhos[,d], and Tsuyoshi Michinobu*[,a]*

[a] Department of Materials Science and Engineering, Institute of Science Tokyo, 2-12-1 Ookayama, Meguro-ku, Tokyo 152-8552, Japan

[b] Department of Forest Resource Chemistry, Forestry and Forest Products Research Institute, Tsukuba, Ibaraki 305-8687, Japan.

[c] Department of Materials Science and Bioengineering, Nagaoka University of Technology, Nagaoka, Niigata 940-2188, Japan.

[d] Department of Chemical Science and Engineering, Institute of Science Tokyo, 2-12-1 Ookayama, Meguro-ku, Tokyo 152-8550, Japan.

*Corresponding author. E-mail: manzhos.s.aa@m.titech.ac.jp (S.M.), michinobu.t.aa@m.titech.ac.jp (T.M.)

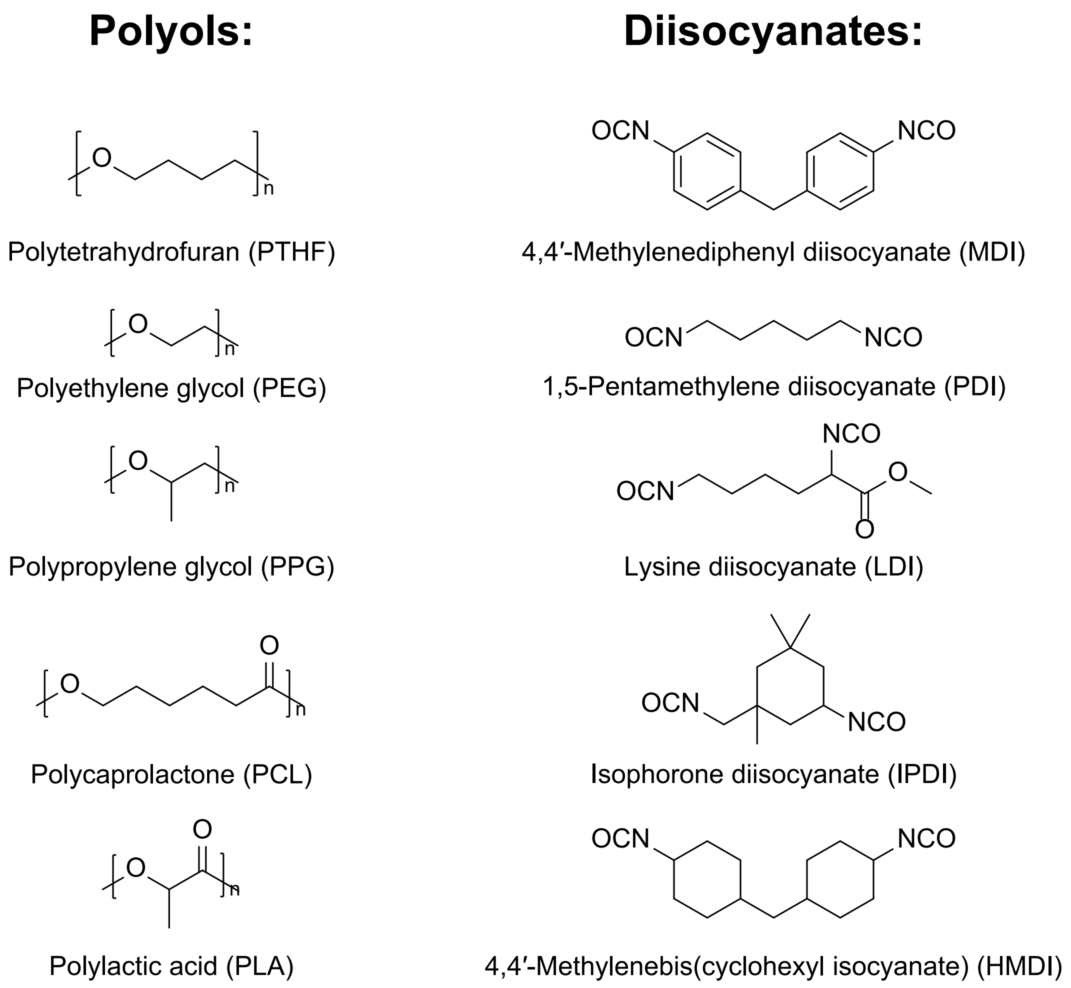

Figure S1. Chemical structures of polyols and diisocyanates.

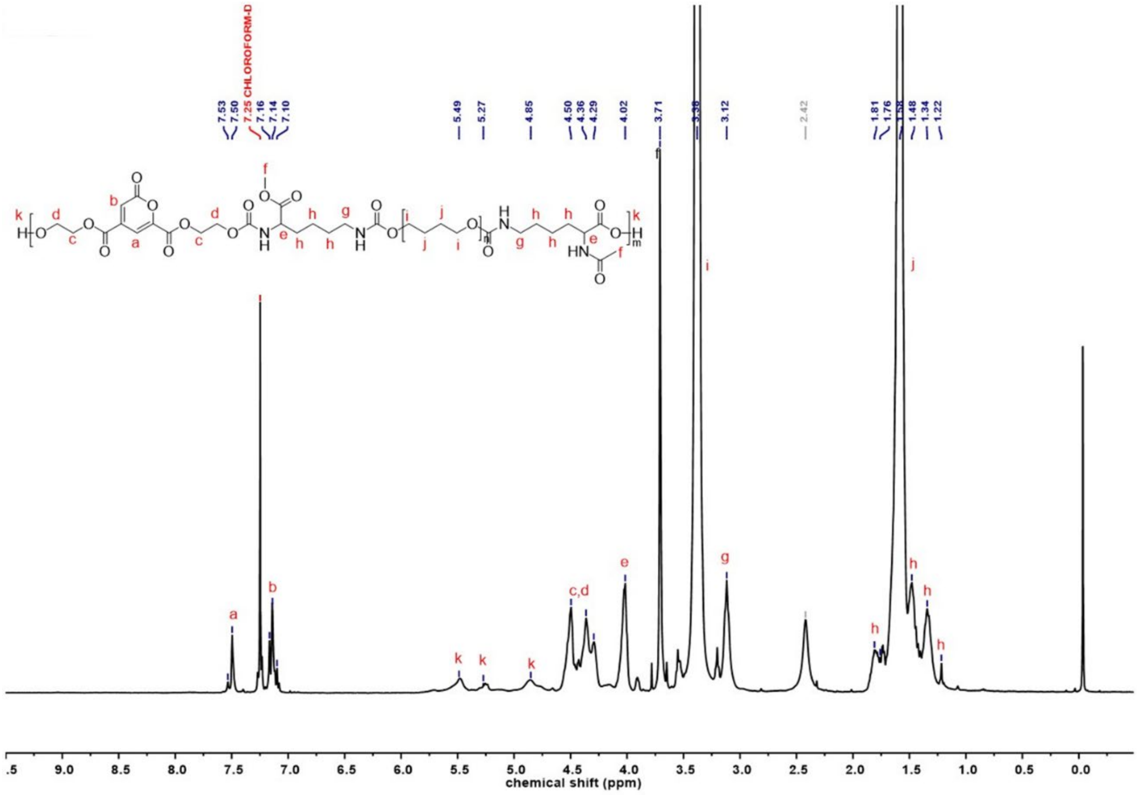
Figure S2. ^{1}H NMR spectrum (400 MHz, $CDCl_3$, 298 K) of No. 3 in **Table 2**.

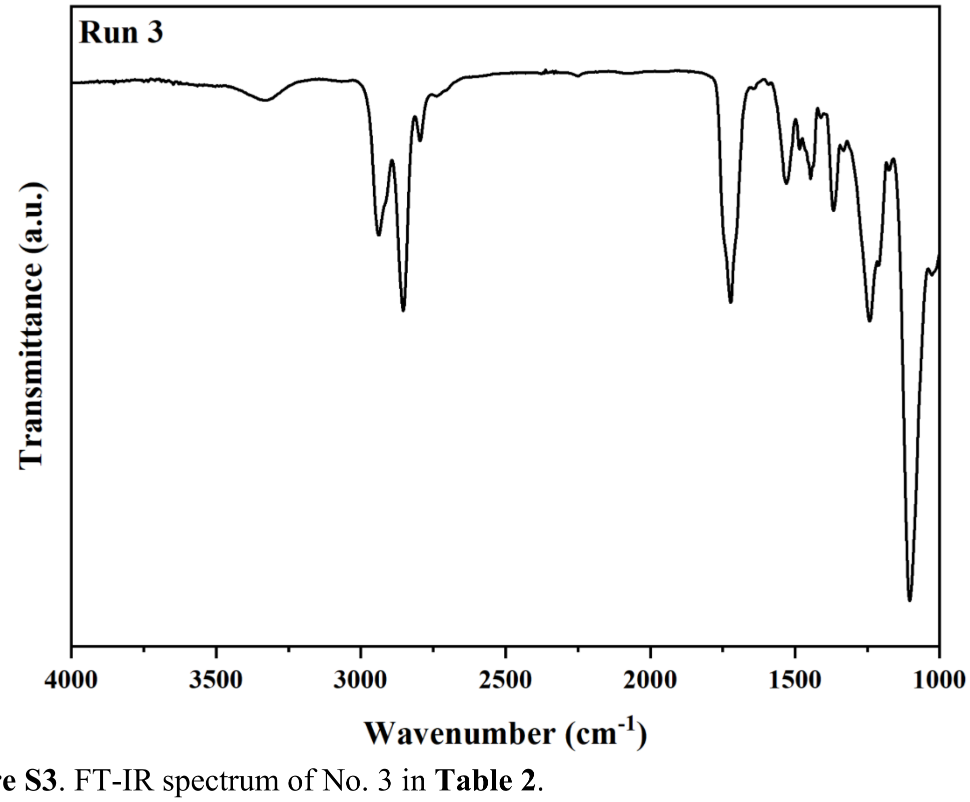

Figure S3. FT-IR spectrum of No. 3 in **Table 2**.

Table S1. The hyperparameters used in ML approaches. The values are optimized with GPyOpt.

ML approach	Hyperparameter
BO	acquisition_type = EI, f = None, model_type = GP, normalize_Y = False, exact_feval = True, domain is set to the same as Table 1, constraints is set to only propose one polyol and one isocyanate
GPR	Length parameter = 0.38<br>Variance = 5.95<br>Noise parameter = 1×10^{-6}
Lasso regression	alpha = 0.0718
Ridge regression	alpha = 4.9301
Random Forest	random_state = 2777, n_estimators =24, max_depth = 5, min_samples_split = 2, min_samples_leaf = 1, max_features = 3, bootstrap =False
Gradient Boosted Decision Tree	n_estimators = 12, learning_rate = 0.3, max_depth = 3, gamma = 0.3, min_child_weight = 3

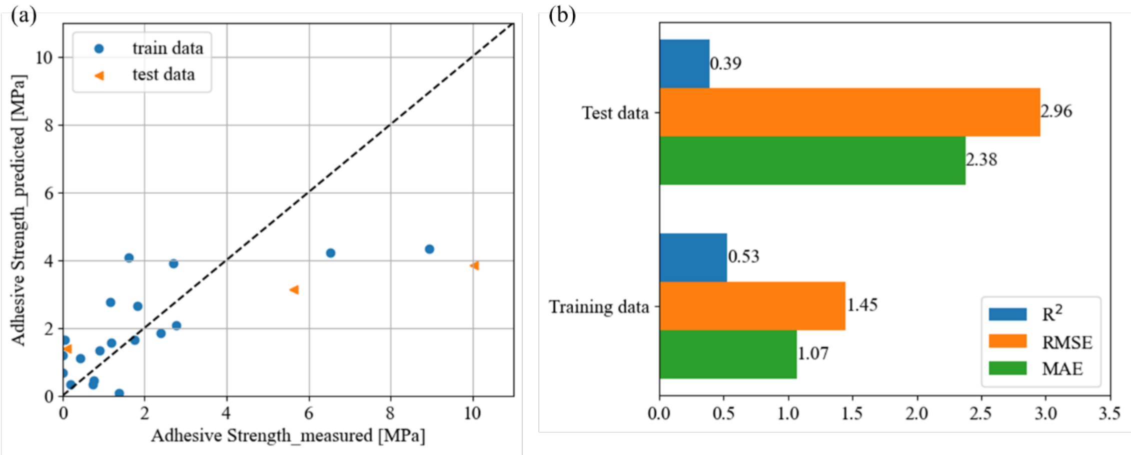

Figure S4. (a) Predicted versus measured adhesive strengths (MPa) from single-lap shear tests when using standard linear regression. The dashed straight line indicates that the predicted and measured adhesive strengths are equal. (b) A comparison of the coefficients of determination (R^2), Root Mean Squared Error (RMSE) and Mean Absolute Error (MAE) for both training and test data.

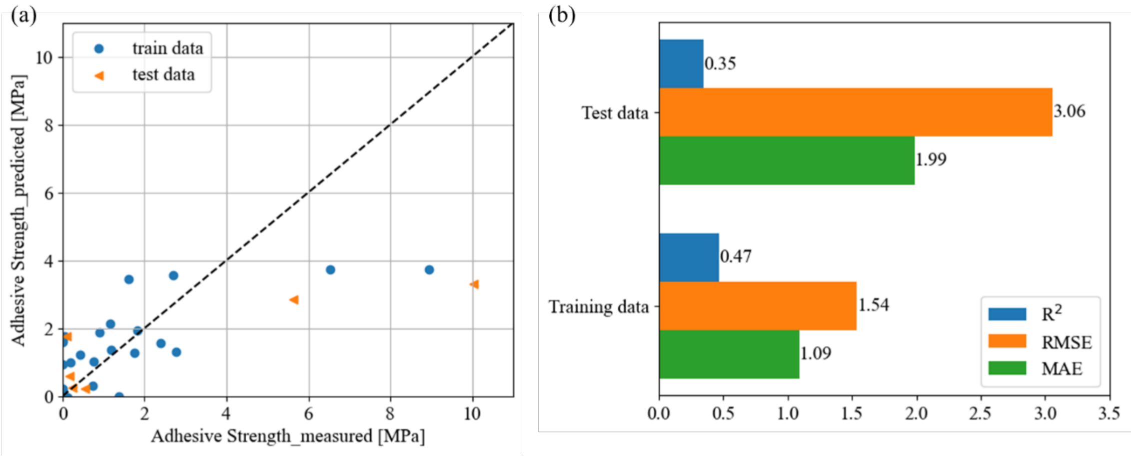

Figure S5. (a) Predicted versus measured adhesive strengths (MPa) from single-lap shear tests when using lasso regression. The dashed straight line indicates that the predicted and measured adhesive strengths are equal. (b) A comparison of the coefficients of determination (R^2), Root Mean Squared Error (RMSE) and Mean Absolute Error (MAE) for both training and test data.

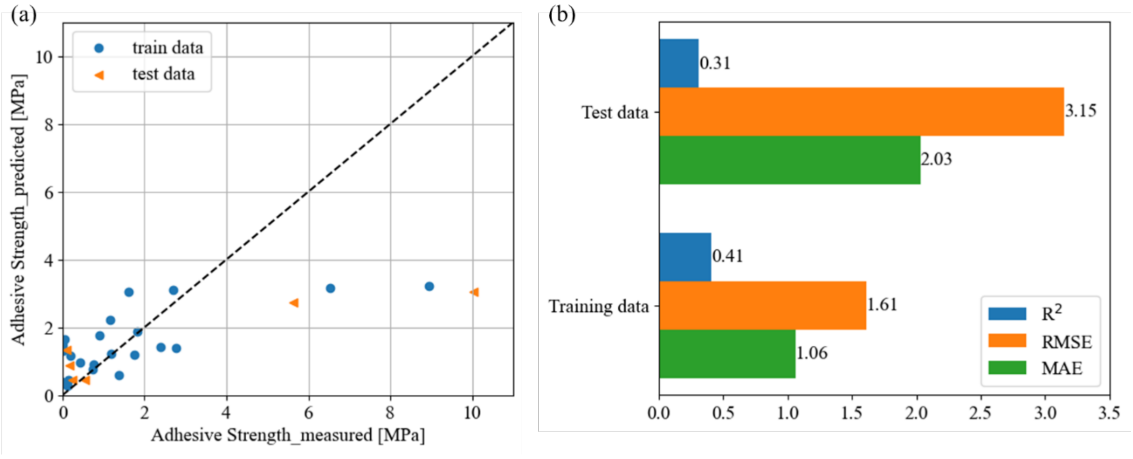

Figure S6. (a) Predicted versus measured adhesive strengths (MPa) from single-lap shear tests when using ridge regression. The dashed straight line indicates that the predicted and measured adhesive strengths are equal. (b) A comparison of the coefficients of determination (R^2), Root Mean Squared Error (RMSE) and Mean Absolute Error (MAE) for both training and test data.

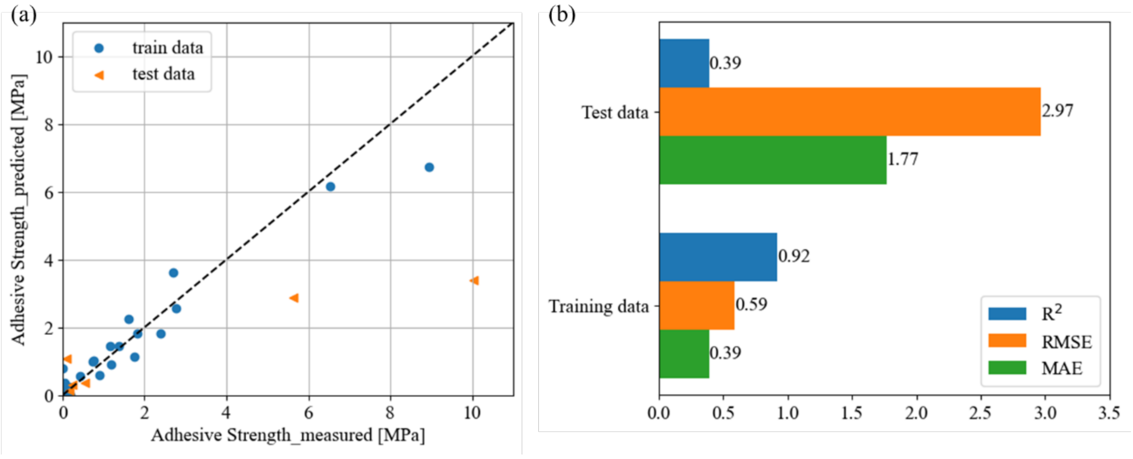

Figure S7. (a) Predicted versus measured adhesive strengths (MPa) from single-lap shear tests when using Gradient Boosted Decision Tree. The dashed straight line indicates that the predicted and measured adhesive strengths are equal. (b) A comparison of the coefficients of determination (R^2), Root Mean Squared Error (RMSE) and Mean Absolute Error (MAE) for both training and test data.

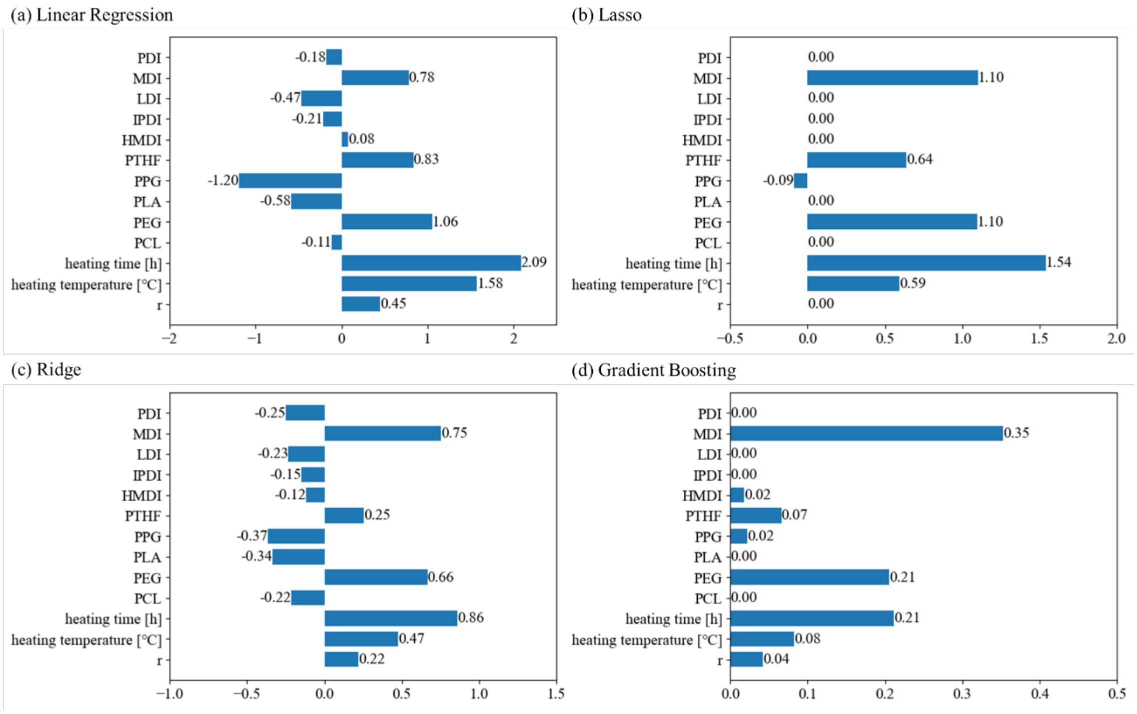

Figure S8. The coefficients in (a) standard linear regression, (b) lasso regression, (c) ridge regression and (d) the feature importance in the Gradient Boosted Decision Treemodel.

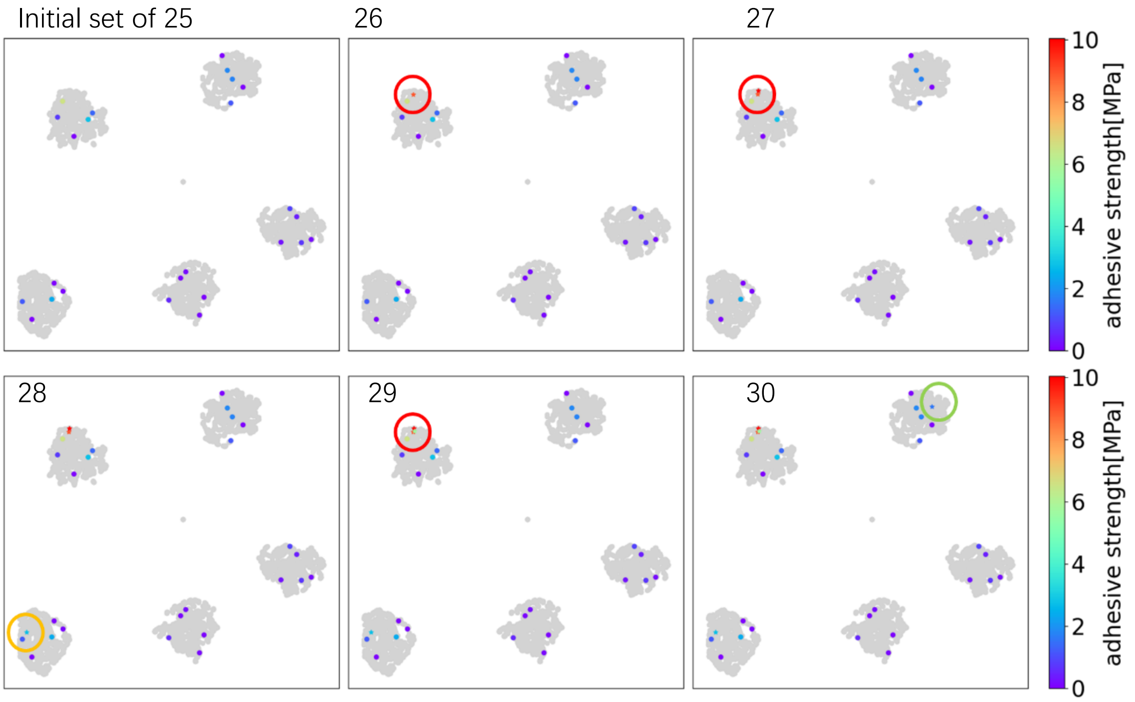

Figure S9. UMAP (Uniform Manifold Approximation and Projection) visualizing clustering patterns in the feature space along BO cycles. Set size of 25 samples corresponds to the 1st cycle, 6 to the 2nd, and so on.

The above UMAP (Uniform Manifold Approximation and Projection) (Figure S9) plot was used to visualize clustering patterns in the feature space along BO cycles. Sample No. 26 and sample No. 10 (that had the highest adhesive strength value in the initial dataset) are quite close in the visualized space, which suggests that the BO iterations are sampling from similar regions. Furthermore, No. 27, which exhibited the best performance in terms of adhesive strength, was also located in this region. On the other hand, sample No. 28 was found to be located farther from this cluster (yellow circle) and had lower adhesive strength (2.71±0.63 MPa). No. 29, after an additional iteration, clustered back close to the high-performing samples (No. 10, 26, and 27). Sample No. 30, however, was farther from this cluster (green circle) and exhibited an even lower adhesive strength (1.6±0.11 MPa). Based on these visualizations, we concluded that the higher-performing samples tended to cluster in the region marked by the red circle. Given that the BO process had led to optimal performance in this region, and the need to manage experimental costs, we decided to terminate the BO loop.
